# Supplementary material for: Structure prediction of protein-ligand complexes from sequence information with Umol
Source: Nat Commun. 2024 May 28;15:4536. doi: 10.1038/s41467-024-48837-6 (PMC11133481; doi:10.1038/s41467-024-48837-6)
Supplement: Supplementary file 1 — Supplementary Information [file 41467_2024_48837_MOESM1_ESM.pdf]

Supplementary Information

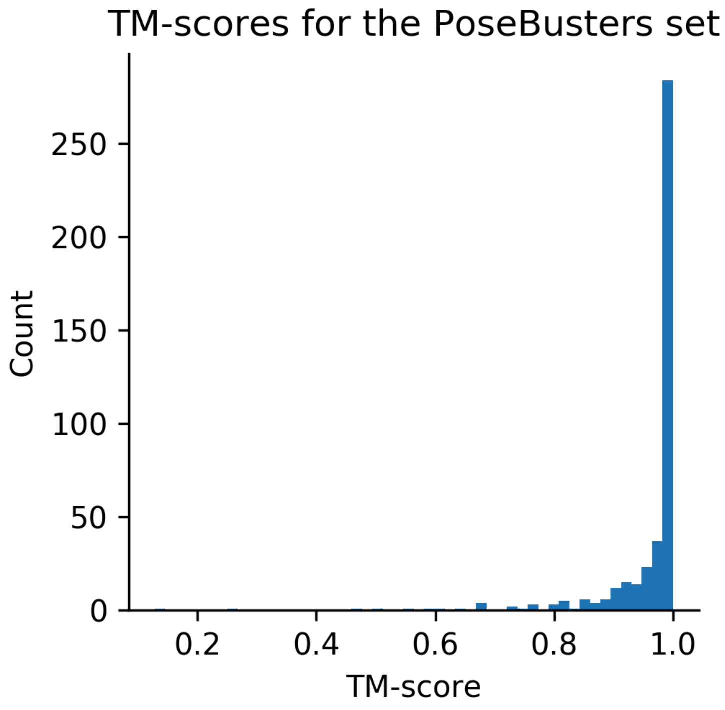

Supplementary Figure 1. TM-scores for the PoseBusters test set comparing the predicted and native protein structures (n=428). The average TM-score is 0.96.

Supplementary Note 1. What determines the ligand RMSD?

Targets with higher sequence identity to the training set tend to be predicted with higher accuracy (Supplementary Table 1), but this does not fully explain the performance difference between targets. To see if certain features make the predictions more accurate, we analyse the conformer error when comparing the distance matrix of the ligand generated with RDKit (Methods) vs the predicted atomic positions, the ligand size, RMSD of the protein pocket atoms, the number of pocket residues, the number of effective sequences (Methods) and the protein length. We find only weak correlations among all features (**Supplementary Figure 2**), with the highest (Spearman R=0.56) being for the conformer error and the lowest for the protein length (Spearman R=0).

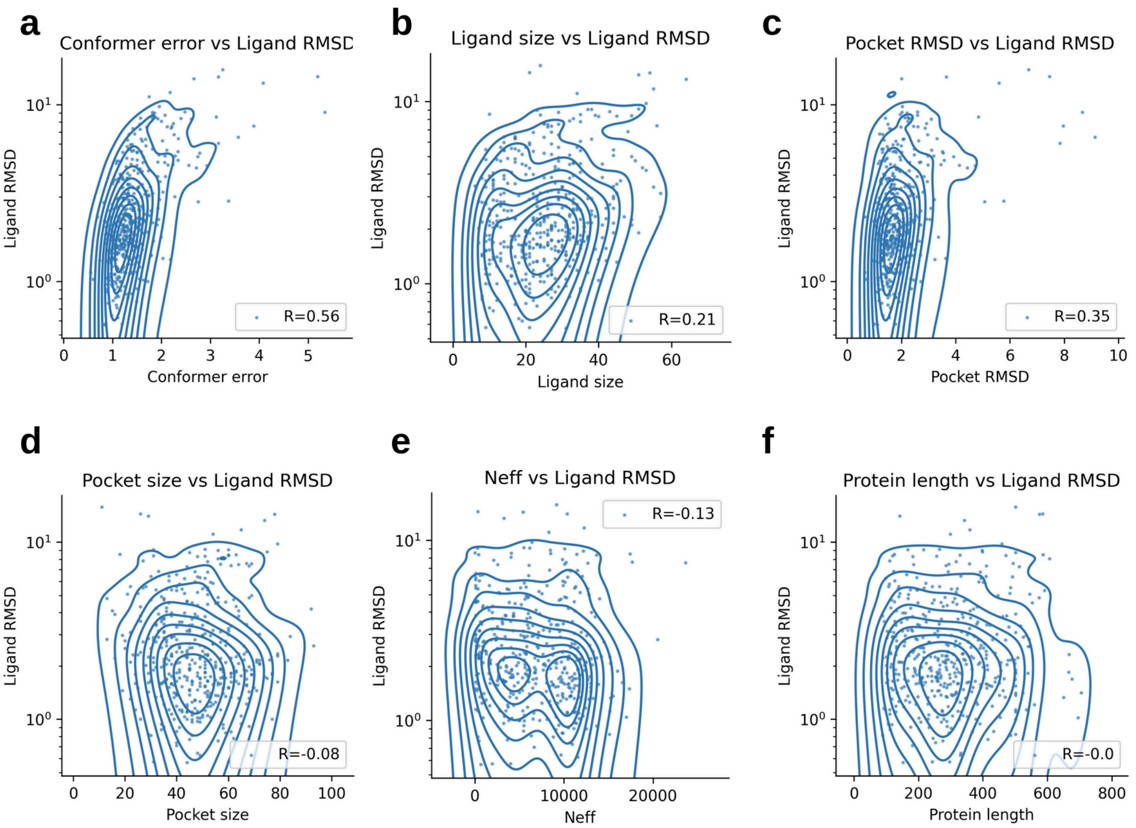

Supplementary Figure 2. Analysis of the relationship between different features and ligand RMSD (LRMSD) for the predictions on the PoseBusters test set (n=428) with Umol-pocket. The lines represent the density of the data points (dots). **a)** The error between the predicted constraints and the ligand conformer generated by RDKit (Conformer error) vs the LRMSD. **b)** Ligand size (number of atoms) vs LRMSD. **c)** RMSD of all the atoms in the protein pocket vs the LRMSD. **d)** The number of pocket residues vs the LRMSD. **e)** The number of effective sequences vs the LRMSD. **f)** The protein length (number of amino acids) vs the LRMSD.

Supplementary Note 2. Difficult targets

To visualise what may distinguish complexes that are predicted well to those that are not, we select the bottom five complexes in terms of average LRMSD across all methods. We focus on targets with <30% sequence identity to PDBBind to adjust for overfitting. The bottom five complexes (highest LRMSD) are PDB IDs 8E77, 7QE4, 8F4J, 7MSR and 7JNB with Umol LRMSDs 11.74, 1.59, 13.31, 2.33 and 2.52 respectively (Figure 3). 7EQ4 is successful at 2 Å LRMSD and 7MSR and 7JNB are close suggesting that Umol-pocket can find correct poses even when other methods struggle. The larger, more complicated ligands 8W77 and 8F4J are predicted in the wrong orientations, suggesting that larger ligands are more difficult to dock. However, this relationship is not apparent when analysing all ligands (Supplementary Figure 2).

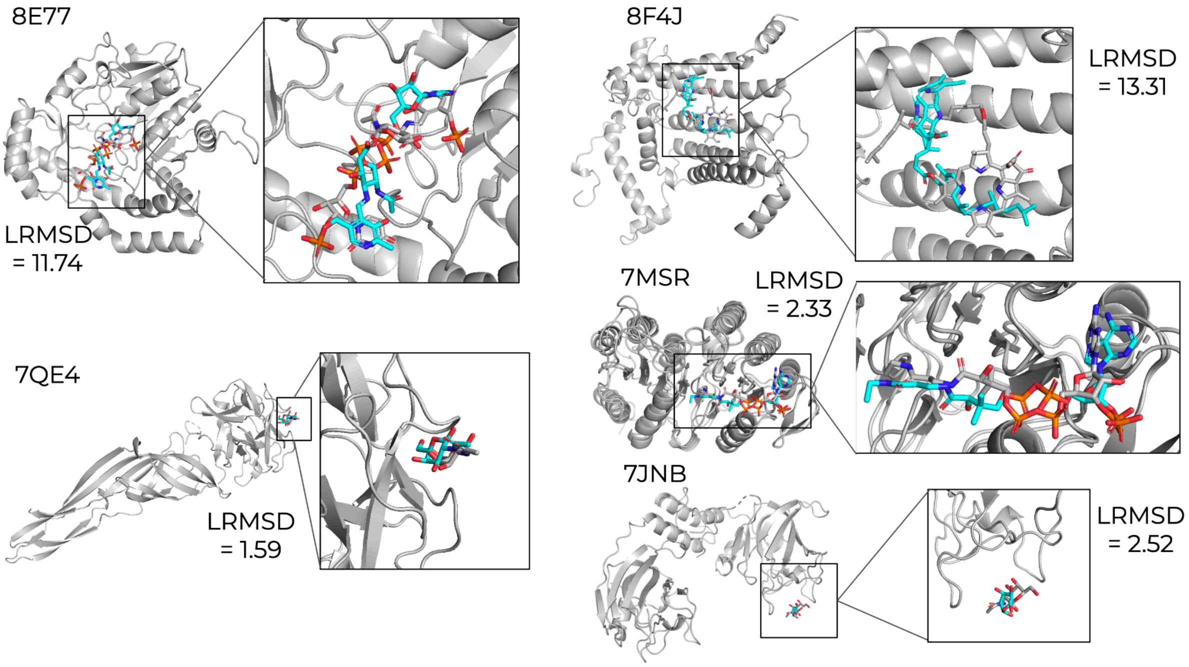

Supplementary Figure 3. The 5 most difficult structures in terms of average LRMSD across all methods are 8E77, 7QE4, 8F4J, 7MSR and 7JNB with Umol ligand RMSDs 11.74, 1.59, 13.31, 2.33 and 2.52 respectively. The native structures are in grey and the predicted ligands in cyan.

Supplementary Note 3. Tanimoto similarity and ligand RMSD

To calculate the Tanimoto similarity coefficient [41] we first computed the 2048-bit Morgan fingerprints [42] with a radius of 2 for each ligand SMILES string in both the PoseBusters and training datasets using RDKit (version 2023.03.2, https://www.rdkit.org). We then computed the pairwise Tanimoto similarity between the two datasets and selected the closest neighbour of each PoseBusters ligand in the training set, the distribution for which is shown in Supplementary Figure 4a. Approximately 14% of the test ligands had a near-identical match in the training dataset. To assess how this overlap between the datasets may affect model success, we also show the relationship between ligand RMSD and Tanimoto similarity to the training set for the PoseBuster's set using Umol-pocket (Supplementary Figure 4b). As found in previous studies [13,14] the ligand similarity is not biassing the outcome. Instead, the protein seems to be what is the cause of overfitting (Supplementary Table 1).

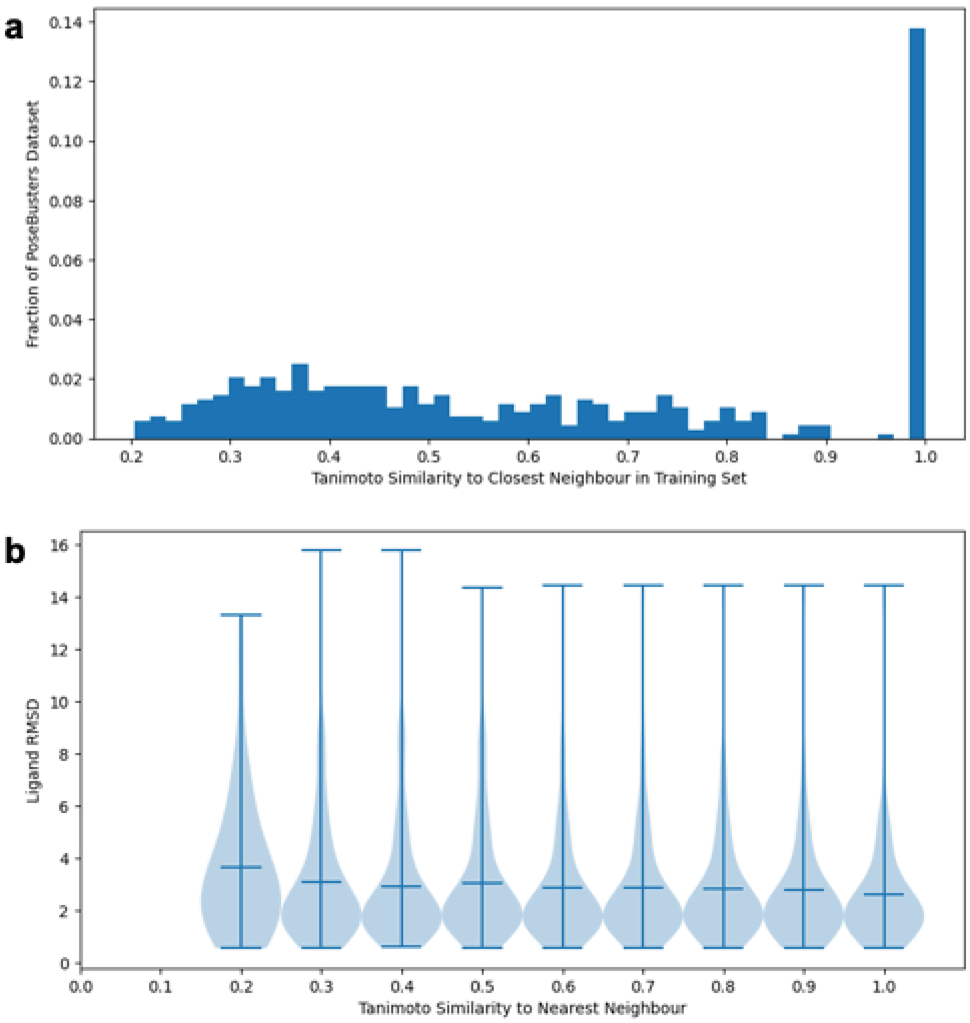

Supplementary Figure 4. a) Pairwise Tanimoto similarity between PoseBusters (n=428) and training datasets (n=16420) for each molecule across the datasets. b) Distribution of ligand RMSD for the PoseBuster's set (n=428) using Umol-pocket across the range of Tanimoto similarity values.

Supplementary Note 4. ROC AUC for selecting high-affinity binders from BindingDB

To see how well high-affinity binders (<20 nM) can be selected, we divided all BindingDB predictions into those that have plDDT <50 or >80. We then create a ROC curve [43] based on selecting binders <20nM. The Corresponding AUC values are 0.66, 0.60, 0.55 and 0.57 for Kd, Ki, EC50 and IC50 data, respectively. A random selection would have an AUC of 0.5, suggesting that the ligand plDDT is informative even without pocket information available.

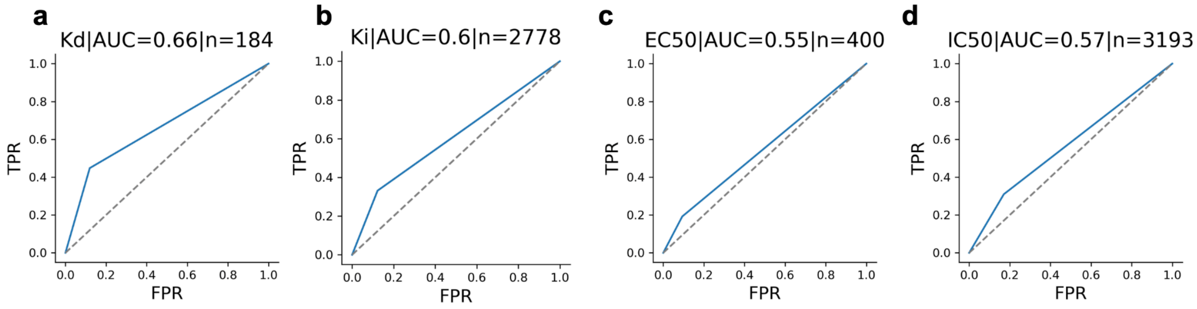

Supplementary Figure 5. ROC curves for affinity data from BindingDB (n=27810). Predictions that have plDDT <50 or >80 are included. We then create an ROC curve based on selecting binders <20nM. The Corresponding AUC values are 0.66, 0.60, 0.55 and 0.57 for Kd, Ki, EC50 and IC50 data, respectively (a-d). The grey dashed line represents the random performance. We also calculate the p-values between the affinity distributions using a selection of plDDT <50 or >80. The Corresponding p-values (one-sided t-test associating having a higher affinity value with a lower plDDT) are 1.58e-17, 5.45e-18, 0.0052 and 0.059 for Kd, Ki, EC50 and IC50 data, respectively (a-d).

Supplementary Table 1. Success rate (% with ligand RMSD≤2Å) on the PoseBuster benchmark set divided by sequence identity (seqid) to the PDBBind 2020 release. The number of proteins with ≥30% seqid is 300 and <30% seqid 128 (n=428 in total). For Vina and Gold the performance remains high below 30% seqid. For diffdock, the performance is much lower below 30% seqid. Umol-pocket has the highest performance of the AI-based methods, also <30% seqid (35.2%). Umol without pocket information performs poorly <30% seqid (4.7%). NeuralPlexer1 has similar performance (23-24%) above/below 30% seqid. For RFAA, the SR is higher <30% seqid which suggests potential data leakage between train and test sets.

**Method**	**SR <30% seqid (n=128)**	**SR ≥30% seqid (n=300)**	**Overall SR (n=428)**
AutoDock Vina [7]	0.517	0.527	0.523
Gold [36]	0.504	0.520	0.512
Umol-pocket	0.352	0.497	0.453
Umol	0.047	0.243	0.185
RFAA [14]	0.516	0.373	0.420
RFAA w/o templates [14]	0.094	0.077	0.082
NeuralPlexer1 [19]	0.234	0.243	0.241
DiffDock [2]	0.148	0.480	0.379
AF+DiffDock [2,15]	0.055	0.283	0.215
Uni-Mol [38]	0.211	0.237	0.229
DeepDock [31]	0.117	0.204	0.178
TANKBind [4]	0.016	0.207	0.150
EquiBind [5]	0.000	0.037	0.026
