# Supplementary material for: Structure prediction of protein-ligand complexes from sequence information with Umol
Source: Nat Commun. 2024 May 28;15:4536. doi: 10.1038/s41467-024-48837-6 (PMC11133481; doi:10.1038/s41467-024-48837-6)
Supplement: Supplementary file 2 — Reporting Summary [file 41467_2024_48837_MOESM2_ESM.pdf]

nature portfolio

Corresponding author(s): Patrick Bryant

Last updated by author(s): Nov 14, 2023

Reporting Summary

Nature Portfolio wishes to improve the reproducibility of the work that we publish. This form provides structure for consistency and transparency in reporting. For further information on Nature Portfolio policies, see our Editorial Policies and the Editorial Policy Checklist.

Statistics

For all statistical analyses, confirm that the following items are present in the figure legend, table legend, main text, or Methods section.

n/a	Confirmed	
☐	☒	The exact sample size (*n*) for each experimental group/condition, given as a discrete number and unit of measurement
☐	☒	A statement on whether measurements were taken from distinct samples or whether the same sample was measured repeatedly
☐	☒	The statistical test(s) used AND whether they are one- or two-sided<br>*Only common tests should be described solely by name; describe more complex techniques in the Methods section.*
☐	☒	A description of all covariates tested
☐	☒	A description of any assumptions or corrections, such as tests of normality and adjustment for multiple comparisons
☐	☒	A full description of the statistical parameters including central tendency (e.g. means) or other basic estimates (e.g. regression coefficient) AND variation (e.g. standard deviation) or associated estimates of uncertainty (e.g. confidence intervals)
☒	☐	For null hypothesis testing, the test statistic (e.g. *F*, *t*, *r*) with confidence intervals, effect sizes, degrees of freedom and *P* value noted<br>*Give P values as exact values whenever suitable.*
☒	☐	For Bayesian analysis, information on the choice of priors and Markov chain Monte Carlo settings
☒	☐	For hierarchical and complex designs, identification of the appropriate level for tests and full reporting of outcomes
☒	☐	Estimates of effect sizes (e.g. Cohen's *d*, Pearson's *r*), indicating how they were calculated

Our web collection on statistics for biologists contains articles on many of the points above.

Software and code

Policy information about availability of computer code

Data collection	The software used is described throughout the methods section. Here are the versions:<br>HH-suite version 3.1.0 - MSA generation<br>MMseqs2 version f5f780acd64482cd59b46eae0a107f763cd17b4d - sequence clustering
Data analysis	Python version 3 - visualization and calculation of success rates, etc.<br>TMalign version 20220412 - scoring of predicted proteins

For manuscripts utilizing custom algorithms or software that are central to the research but not yet described in published literature, software must be made available to editors and reviewers. We strongly encourage code deposition in a community repository (e.g. GitHub). See the Nature Portfolio guidelines for submitting code & software for further information.

Data

Policy information about availability of data

All manuscripts must include a data availability statement. This statement should provide the following information, where applicable:

- Accession codes, unique identifiers, or web links for publicly available datasets
- A description of any restrictions on data availability
- For clinical datasets or third party data, please ensure that the statement adheres to our policy

All data needed to reproduce this study, including results and scoring metrics are available through zenodo: https://zenodo.org/records/10060894
We will update this repository to include all additional analyses included in the study.
The original data used for training can also be extracted from PDBbind: http://www.pdbbind.org.cn
The PoseBusters test set is available at: https://zenodo.org/record/8278563
All data is freely and publicly available

Human research participants

Policy information about studies involving human research participants and Sex and Gender in Research.

Reporting on sex and gender	NA
Population characteristics	NA
Recruitment	NA
Ethics oversight	NA

Note that full information on the approval of the study protocol must also be provided in the manuscript.

Field-specific reporting

Please select the one below that is the best fit for your research. If you are not sure, read the appropriate sections before making your selection.

- [x] Life sciences
- [ ] Behavioural & social sciences
- [ ] Ecological, evolutionary & environmental sciences

For a reference copy of the document with all sections, see nature.com/documents/nr-reporting-summary-flat.pdf

Life sciences study design

All studies must disclose on these points even when the disclosure is negative.

Sample size	All publicly available data from the PDB processed in PDBBind was used.
Data exclusions	A few examples could not be processed due to ambiguous ligand stereochemistry or missing protein residue information. This is described in the methods section.
Replication	All data - including processed versions for training and evaluation - is available through the linked zenodo repositories.
Randomization	The protein sequences from PDBBind were clustered on 20% sequence identity and the data was split based on these clusters for training, validation and affinity evaluation. The PoseBusters test set contains data based on a date split towards PDBBind. This data was further divided on sequence identity (30%) according to the authors' study.
Blinding	NA. Since we split the sequence clusters randomly and used a test set created in an independent study, blinding is not applicable.

Reporting for specific materials, systems and methods

We require information from authors about some types of materials, experimental systems and methods used in many studies. Here, indicate whether each material, system or method listed is relevant to your study. If you are not sure if a list item applies to your research, read the appropriate section before selecting a response.

Materials & experimental systems

n/a	Involved in the study
☒	☐ Antibodies
☒	☐ Eukaryotic cell lines
☒	☐ Palaeontology and archaeology
☒	☐ Animals and other organisms
☒	☐ Clinical data
☒	☐ Dual use research of concern

Methods

n/a	Involved in the study
☒	☐ ChIP-seq
☒	☐ Flow cytometry
☒	☐ MRI-based neuroimaging
